# Perspectives on ROCK2 as a Therapeutic Target for Alzheimer’s Disease

**DOI:** 10.3389/fncel.2021.636017

**Published:** 2021-03-15

**Authors:** Audrey J. Weber, Jeremy H. Herskowitz

**Affiliations:** Center for Neurodegeneration and Experimental Therapeutics, Departments of Neurology and Neurobiology, University of Alabama at Birmingham, Birmingham, AL, United States

**Keywords:** ROCK2, Alzheimer’s disease, tau, autophagy, mTOR, dendritic spine

## Abstract

Rho-associated coiled-coil containing kinase isoform 2 (ROCK2) is a member of the AGC family of serine/threonine kinases and an extensively studied regulator of actin-mediated cytoskeleton contractility. Over the past decade, new evidence has emerged that suggests ROCK2 regulates autophagy. Recent studies indicate that dysregulation of autophagy contributes to the development of misfolded tau aggregates among entorhinal cortex (EC) excitatory neurons in early Alzheimer’s disease (AD). While the accumulation of tau oligomers and fibrils is toxic to neurons, autophagy facilitates the degradation of these pathologic species and represents a major cellular pathway for tau disposal in neurons. ROCK2 is expressed in excitatory neurons and pharmacologic inhibition of ROCK2 can induce autophagy pathways. In this mini-review, we explore potential mechanisms by which ROCK2 mediates autophagy and actin dynamics and discuss how these pathways represent therapeutic avenues for Alzheimer’s disease.

## Introduction

The small Rho guanosine triphosphatase (GTPase) protein RhoA is a key regulator of various cellular processes, including actomyosin dynamics, cell proliferation, survival, and gene expression (Jaffe and Hall, 2005). The primary downstream targets of GTP-bound activated RhoA are the Rho-associated coiled-coil kinase (ROCK) isoforms 1 and 2. ROCK1 and ROCK2 are serine/threonine kinases that share 65% similarity in their amino acid sequence with 95% similarity in their kinase domains (Nakagawa et al., 1996). Both ROCK isoforms display mostly similar expression patterns in mammals, with higher transcript levels of ROCK1 in the thymus and blood, and ROCK2 in the brain (Julian and Olson, 2014). ROCKs phosphorylate various substrates that heavily influence cellular morphology, adhesion, and motility. Through these mechanisms, ROCK1 and ROCK2 are considered therapeutic targets for cancer, asthma, insulin resistance, kidney failure, osteoporosis, and erectile dysfunction (Olson, 2008; Schaafsma et al., 2008; Lee et al., 2009; Albersen et al., 2010; Komers et al., 2011; Rath and Olson, 2012). In addition, ROCKs are implicated in a number of neurodegenerative disorders, including spinal cord injury, stroke, glaucoma, Alzheimer’s disease (AD), Frontotemporal Dementia (FTD), Parkinson’s disease, and Amyotrophic Lateral Sclerosis (ALS; Shibuya et al., 2005; Duffy et al., 2009; Herskowitz et al., 2011, 2013; Challa and Arnold, 2014; Koch et al., 2014; Gentry et al., 2016; Henderson et al., 2016; Tatenhorst et al., 2016; Gunther et al., 2017). This mini review will focus on ROCK2 and its emerging role as an autophagy regulatory protein, which may provide multiple beneficial effects for AD intervention.

## Rock2 Signaling Protects Dendritic Spines in Alzheimer’S Disease

Dendritic spines are actin-rich protrusions along dendrites that formulate the postsynaptic sites of the majority of excitatory synapses in the brain. Spines are critical for proper neuronal function and exhibit remarkable variability in size and shape. ROCKs are well-studied regulators of actin-myosin-mediated cytoskeleton contractility, and recent work has provided evidence for ROCK isoform-specific effects on dendritic spine morphology. Two studies indicated that ROCK1 influences dendritic spine length through mechanisms involving actin-myosin pathways (Newell-Litwa et al., 2015; Henderson et al., 2019). In contrast, ROCK2 mediates spine density through the serine/threonine LIM domain kinase (LIMK) isoform 1 and subsequent phosphorylation of cofilin (Henderson et al., 2019).

Cognitive impairment in AD is the result of synapse loss in brain regions that are critical for memory processes. Synapse or dendritic spine loss correlates more strongly with cognitive impairment in AD than amyloid-β (Aβ) or neurofibrillary tangle (NFT) pathology (Braak and Braak, 1991; Scheff et al., 2006; Boros et al., 2017, 2019). This suggests that druggable targets, like ROCK2, that regulate spine density and morphology are avenues for AD therapeutics. To this end, Aβ can induce dendritic spine degeneration by activating RhoA and ROCK2. Increased activity of ROCK2 has detrimental consequences on dendritic spine structure and function in model systems (Swanger et al., 2015; Sellers et al., 2018; Henderson et al., 2019). Moreover, ROCK2 protein levels are increased among mild cognitive impairment (MCI) and AD patients, suggesting that ROCK2 may contribute to synaptic loss in early disease stages (Herskowitz et al., 2013). Preclinical studies demonstrated that treatment of hippocampal neurons with the pan-ROCK inhibitor Fasudil or an experimental LIMK1 inhibitor blocked Aβ-induced spine degeneration and neuronal hyperexcitability (Rush et al., 2018; Henderson et al., 2019). These findings highlighted the ROCK2-LIMK1 pathway as a therapeutic target to provide dendritic spine resilience against Aβ, which could benefit cognitively normal patients that are at high risk for developing AD dementia. However, ROCK2 is also implicated in the intracellular degradation of the microtubule-associated protein tau via autophagic pathways. This broadens the potential benefit of ROCK2-based therapies for AD, which we will discuss later in the review.

## Mtor as A Regulator of Autophagy

In eukaryotic cells, autophagy and the proteasome are major degradation pathways for intracellular debris (Mizushima and Komatsu, 2011). Autophagy is a lysosome-mediated intracellular pathway in which cytoplasmic material is delivered to and degraded in the lysosome. A major function of autophagy is to maintain quality control over proteins and organelles. Autophagy can remove long-lived, aggregated, and/or misfolded proteins, as well as regulate cellular differentiation, defense against pathogens, and nutritional starvation (Ravanan et al., 2017). Currently, three types of autophagy are known to occur in eukaryotic cells: microautophagy, chaperone-mediated autophagy, and macroautophagy. Macroautophagy is hypothesized to be the primary pathway for the delivery of cargoes to the lysosome for degradation and recycling (Hara et al., 2006; Yang and Klionsky, 2010). Henceforth, we refer to macroautophagy as “autophagy”.

Autophagy uses the intermediate organelle, the autophagosome, to shuttle cytoplasmic debris to the lysosome. First, an isolation membrane, known as the phagophore, engulfs soluble materials and organelles, thereby forming the autophagosome. The autophagosome then fuses with the lysosome to become an autolysosome and degrades materials engulfed within it (Hara et al., 2006). The mammalian target of rapamycin (mTOR) is a primary upstream regulator of autophagy. mTOR is a serine/threonine kinase that forms the catalytic subunit of two distinct protein complexes, known as mTOR Complex 1 (mTORC1) and 2 (mTORC2) (Hara et al., 2006). mTORC1 has five components: mTOR, the regulatory-associated protein of mTOR, mammalian lethal with SEC13 protein 8, proline-rich AKT1 substrate 1, and DEP domain-containing mTOR-interacting protein. mTORC2 is composed of mTOR, rapamycin-insensitive companion of mTOR (Rictor), mammalian lethal with SEC13 protein 8, and mammalian stress-activated protein kinase interacting protein 1 (Lipton and Sahin, 2014).

The induction of autophagosome biogenesis is regulated by autophagy-related (Atg) proteins that nucleate the ULK1 complex. The ULK1 complex consists of three regulatory subunits: ATG13, FIP200, and ATG101 (Saxton and Sabatini, 2017). An amino acid-rich cellular environment results in mTORC1 binding and phosphorylating ULK1 at Ser757, resulting in the inhibition of autophagosome formation (Ganley et al., 2009; Jung et al., 2009). During amino acid starvation, mTORC1 dissociates from ULK1, thereby allowing ULK1 activation by AMP-activated protein kinase (AMPK), a key activator of autophagosome formation (Kim et al., 2011). In an alternative pathway, activation and translocation of the transcription factor EB (TFEB) into the nucleus leads to increased expression of genes related to lysosomal biogenesis and autophagy machinery (Martina et al., 2012; Roczniak-Ferguson et al., 2012; Settembre et al., 2012). mTORC1 can also regulate autophagy in part by phosphorylating TFEB. TFEB phosphorylation at Ser211 results in the inactivation of TFEB in the cytoplasm (Martina et al., 2012).

mTORC2 is known to phosphorylate members of the AGC family of protein kinases, including protein kinase B (also known as Akt), protein kinase C, and serum and glucocorticoid-regulated kinase 1 (SGK1) (Saxton and Sabatini, 2017). Phosphorylation of Akt by mTORC2 may play dual roles concerning autophagy induction. One study suggests that phosphorylation of Akt by mTORC2 inhibits the activation of the transcription factor FoxO3, thereby suppressing autophagy-related gene expression (Mammucari et al., 2007). Recent work evaluating loss of mTORC2 activity showed that the downstream effector SGK-1 can inhibit autophagy independent of FoxO3 (Aspernig et al., 2019). Additional work in primary rat hippocampal neurons demonstrated that mTORC2-mediated phosphorylation of Akt can activate mTORC1, thus influencing autophagy through an alternative mechanism (Urbanska et al., 2012). mTORC2-mediated activation of mTORC1 was further confirmed in human cells, which showed a reduction of phosphorylated mTORC1 and p70S6K, indicating mTORC2 can inhibit autophagy through activation of mTORC1 (Huang et al., 2015). Collectively, these studies indicate that both mTORC1 and mTORC2 can modulate autophagy induction. However, mTORC2 is also an intriguing regulator of the actin cytoskeleton, which we will discuss later in the review.

## Autophagy in Alzheimer’S Disease

**Figure 1:**
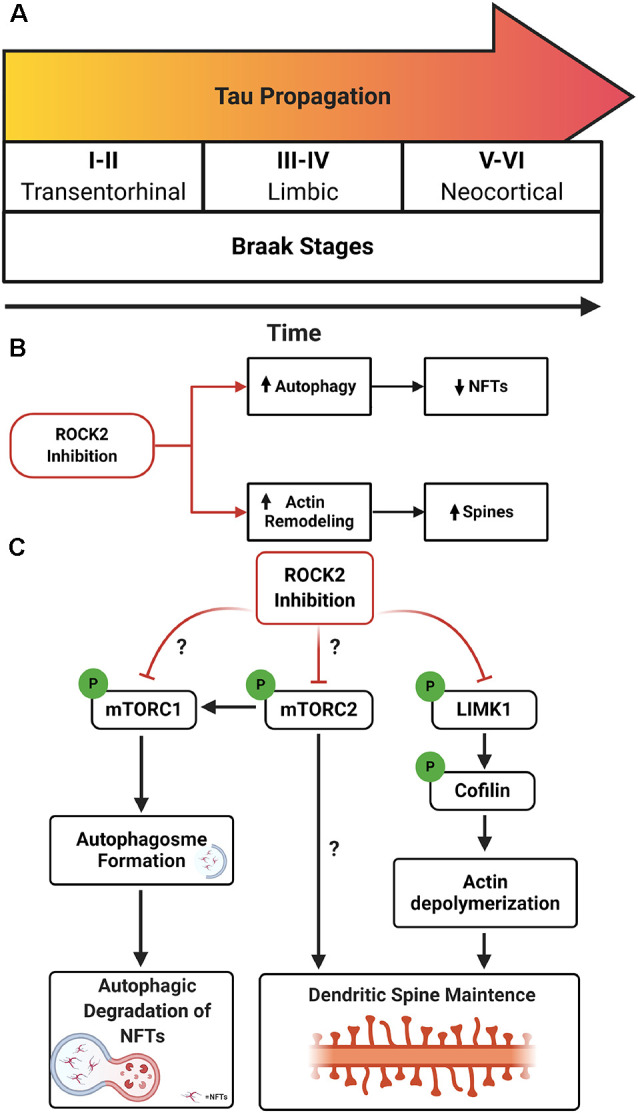
Rho-associated coiled-coil kinase isoforms 2 (ROCK2) inhibition as a therapeutic avenue for Alzheimer’s disease (AD). **(A)** Schematic of Braak stages I–VI which describes the hypothetical spread of neurofibrillary tangles (NFTs) over time in AD. Tau aggregates initiate in the entorhinal cortex (EC; stages I–II) before spreading to limbic regions such as the hippocampus (stages III–IV) and eventually the neocortex (stages V–VI). **(B)** Therapeutic benefits of targeting ROCK2 in AD. Pharmacologic inhibition of ROCK2 enhances autophagic processes, reducing tau protein levels and pathology. In parallel, ROCK2 inhibition can increase actin dynamics, stimulating dendritic spine structural plasticity. **(C)** Mechanisms of ROCK2 therapeutics. (Left to right) ROCK2 inhibition reduces mammalian targetof rapamycin (mTOR) phosphorylation which stimulates autophagosome formation and facilitates tau aggregate degradation. Reducing mTOR phosphorylation could influence the activity of mTORC1 and/or mTORC2. ROCK2 may alter mTORC2’s role in actin cytoskeleton rearrangement. ROCK2 inhibition reduces phosphorylation of LIMK1, inactivating LIMK1. Reduced activity of LIMK1 maintains cofilin activation and actin depolymerization, enhancing structural plasticity of dendritic spines.

AD neuropathology is characterized by the presence of extracellular Aβ deposits and intracellular NFTs comprised of misfolded, hyperphosphorylated tau (Wu et al., 2016; Goedert et al., 2017). The pathological spread of tau aggregates correlates with cognitive decline in AD, suggesting that propagation of NFTs contributes to synapse loss (Braak et al., 2006; Scholl et al., 2016). The spread of tau in AD is hypothesized to operate in a prion-like manner in which tau seeds spread via connected neurons. The tau seed(s) is hypothesized to interact with native tau in the cytosol, drive misfolding of tau, and ultimately generate neurofibrillary pathology (Hallinan et al., 2019). In the early stages of AD, tau aggregates are found in somatodendritic compartments of layer 2/3 neurons in the entorhinal cortex (EC) (Braak and Braak, 1991). It is hypothesized that pathologic tau propagates from the EC to the hippocampus via trans-synaptic connections, where it can spread to limbic regions and ultimately neocortical areas in the same manner (Braak and Braak, 1991; De Calignon et al., 2012; Ahmed et al., 2014; Figure 1A).

The precise molecular and cellular mechanisms of tau propagation remain critical questions in the field, but the selective vulnerability of neurons in EC layer 2/3, the subiculum, CA1 of the hippocampus, and basal forebrain cholinergic neurons likely contributes to the initiation of tau aggregates (Davies and Maloney, 1976; Hyman et al., 1984; Stranahan and Mattson, 2010). A recent study to understand the selective vulnerability of neurons suggests that excitatory neurons, in contrast to inhibitory neurons, harbor a cellular environment of aggregation-prone proteins that is susceptible to dysregulation of protein homeostasis systems (Fu et al., 2019). Co-expression network analysis of single-nucleus RNA-seq datasets identified the autophagy facilitator BCL2-associated athanogene 3 (BAG3) as a protector from tau aggregation. Notably, inhibitory neurons, which are often thought to be resilient to tau aggregation, have high expression of BAG3 in both AD and non-AD cases (Fu et al., 2019).

Dysfunction of autophagy and the lysosome system is hypothesized to contribute to AD progression (Boland et al., 2008). Possession of the ε4 allele of Apolipoprotein E (*APOE4)*, a major genetic risk factor for the development of late-onset AD, was shown to disrupt the endo-lysosomal system in EC neurons (Nuriel et al., 2017). Transcriptomic analysis of *APOE3/4* targeted replacement mice identified upregulation of genes associated with vesicle function including nine Vacuolar H^+^-ATPases (V-ATPases) and Rab GTPases which are vital for early and late endosome function. Moreover, immunohistochemistry of the EC and cingulate cortex of *APOE4/4* targeted replacement mice revealed an increase of early endosomes, which is observed in AD (Nuriel et al., 2017). The dysregulation of autophagy within the EC could facilitate the initial aggregation of tau that ultimately spawns pathologic tau spread to other vulnerable brain regions.

To this end, studies in post-mortem AD patient tissue revealed accumulation of autophagic vacuoles within damaged neurites, suggesting defective lysosomal clearance in the neocortex (Nixon et al., 2005). Other studies indicated an increase in mTOR signaling activity in AD brains (Chang et al., 2002; Peel, 2003; Onuki et al., 2004). Immunoblot findings from Tramutola et al. (2015) revealed that mTOR phosphorylation was increased in both AD and mild cognitive impairment (MCI) cases, but not in preclinical AD brains. Furthermore, two autophagy-related proteins, Beclin1 and LC3, were decreased in both AD and MCI tissue, suggesting that mTOR activity-induced autophagy was impaired (Tramutola et al., 2015). These findings support the hypothesis that an increase in mTOR signaling activity could suppress autophagy-related processes, leading to a failure to clear tau aggregates in vulnerable neurons.

From a preclinical standpoint, rapamycin-induced inhibition of mTOR in the 3xTg-AD mouse model increased the abundance of autophagy-related proteins, while simultaneously reducing both soluble Aβ and tau levels (Caccamo et al., 2010). Recent work in cultured human neurons derived from FTD patients further demonstrated a role for mTOR inhibition as a therapeutic strategy to reduce tau levels (Silva et al., 2020). In this study, small molecular screens were used to identify ATP-competitive mTOR inhibitors that are more selective and potent than rapamycin. Patient-derived human neurons were treated with mTOR inhibitors, resulting in decreased levels of insoluble tau after a single dosage. The treatment had lasting effects by increasing autophagy for 12 days post-drug administration. Furthermore, mTOR inhibitors increased neuronal viability by protecting against tau toxicity and stressors such as Aβ (Silva et al., 2020). Together, these findings suggest that mitigating pathogenic tau levels by activating autophagic pathways is a rational strategy for treating tauopathies, including AD. As discussed earlier in this review, pharmacologic inhibition of ROCK2 signaling pathways can generate dendritic spine resilience to Aβ by stimulating actin cytoskeleton rearrangement. However, ROCK2 inhibition can also reduce tau levels by inducing autophagy. This fascinating combination of beneficial effects highlights the potential of ROCK2-based therapeutics for AD. Yet, important questions remain regarding the potential intersection of these two pathways.

## Rock2 as A Therapeutic Target for Alzheimer’S Disease

Because the spread of NFTs, indicated by increasing Braak stages (Figure 1A), correlates more strongly with cognitive decline in AD than extracellular Aβ pathology, it is hypothesized that propagation of tau aggregates contributes to the dendritic spine or synapse loss (Braak et al., 2006; Scholl et al., 2016). Based on this, therapeutics that curb tau propagation, as well as spine loss, would be considered a rational treatment strategy to delay or prevent AD dementia onset. Notably, the hypotensive effect of orally available pan-ROCK inhibitors is likely linked to ROCK1 inhibition, and therefore ROCK2-specific compounds could offer a less severe drop in blood pressure upon systemic exposure (Defert and Boland, 2017). ROCK2’s emerging role as an autophagy regulatory protein, as well as ROCK2’s established link to actin cytoskeleton dynamics, positions it as an exciting, highly interesting drug target. The pan-ROCK inhibitor Fasudil has been clinically approved to treat hypertension, heart failure, glaucoma, spinal cord injury, and stroke (Feng et al., 2015). Based on the beneficial effects of Fasudil on neuronal plasticity and survival, Fasudil is currently being used in clinical trials for the treatment of ALS (Lingor et al., 2019). These efforts fuel excitement about the potential of ROCK inhibitors for the treatment of other neurodegenerative disorders, including AD.

Aβ, a key pathological hallmark of AD, is generated by sequential proteolytic cleavage of the amyloid precursor protein (APP). Fasudil, as well as the experimental small-molecule inhibitor SR3677, which is more selective to ROCK2, were shown to reduce Aβ production in primary neurons and a mouse model of AD. Cell biological studies in HEK293 cells indicated that treatment with SR3677 increased APP and beta-site cleaving enzyme 1 (BACE1) co-localization with the lysosome marker LAMP1, suggesting that pharmacologic inhibition of ROCK2 promotes traffic of APP and BACE1 to lysosomes (Herskowitz et al., 2013). Further studies indicated that RNAi depletion of ROCK1 or ROCK2 in primary neurons reduced endogenous murine Aβ production, which was likely the result of enhanced lysosomal or autophagic degradation of APP (Henderson et al., 2016).

Several studies indicate that pharmacologic inhibition of ROCKs can induce autophagy in mammalian cells (Bauer et al., 2009; Koch et al., 2014; Gentry et al., 2016). Using RNAi depletion methods, Koch et al. (2014) demonstrated that knockdown of ROCK2 or LIMK1 enhanced neurite outgrowth of rat retinal ganglion cells (RGCs) following rat optic nerve crush. However, knockdown of ROCK2, but not LIMK1, increased survival of RGCs after optic nerve axotomy. Further shRNA-mediated knockdown studies of ROCK2 in primary RGC cultures revealed that depletion of ROCK2 increased protein levels of the microtubule-associated protein 1 light chain 3 (LC3-II), which is an essential component for autophagosome formation. Moreover, SQSTM1/p62, which serves as a link between LC3 and ubiquitinated substrates, was reduced after ROCK2 depletion (Koch et al., 2014). Collectively, these findings suggest that inhibition of ROCK2 kinase activity enhances autophagic flux and that this mechanism does not rely on ROCK2 signaling through LIMK1.

Notably, ROCK1 can play a role in metabolic stress-induced autophagy. ROCK1 activity induces autophagy in HeLa cells by interacting with and phosphorylating Beclin1 at threonine 119, whereas blocking ROCK1 activity with the pan-ROCK inhibitor Y27632 reduced nutritional stress-mediated autophagy (Gurkar et al., 2013). In contrast, genetic deletion of ROCK1 had no effect on mTOR signaling in cardiomyocytes (Shi et al., 2019). These findings suggest that ROCK1’s influence over autophagic pathways may be cell-type specific, and whether these mechanisms exist in brain-derived cells remains to be determined.

Postmortem studies in progressive supranuclear palsy (PSP) and corticobasal degeneration (CBD) patients revealed that ROCK1, ROCK2, mTOR, and p70 S6 kinase (S6K) protein levels are increased significantly in dorsolateral prefrontal cortex tissue samples from PSP and CBD cases compared with controls. Moreover, statistical correlation analysis indicated a strong positive correlation among ROCK1, ROCK2, mTOR, and S6K in PSP and CBD cases, suggesting that these signaling pathways could play a role in tau deposition in disease (Gentry et al., 2016). Notably, postmortem studies on MCI and AD patients indicated ROCK2 protein levels are increased in neurons from the dorsolateral prefrontal cortex among disease cases in comparison to controls (Herskowitz et al., 2013). However, whether similar changes in mTOR and S6K protein levels occur in the prefrontal cortex among all tauopathies, including AD, remains to be determined.

Disposal of tau can also be mediated by autophagy, specifically via mTOR signaling through S6K in neurons (Boland et al., 2008; Kruger et al., 2012). Neurons treated with SR3677 exhibited robust depletion of soluble and insoluble tau protein levels which coincided with reduced mTOR phosphorylation at serine 2448, reduced protein levels of S6K and p62, and increased protein levels of LC3-II (Gentry et al., 2016). These findings indicate that ROCK2 inhibition stimulates autophagy induction in neurons and results in the reduction of tau protein levels. Notably, Gentry et al. also showed that treatment with Fasudil mitigated pathogenic tau levels and suppressed rough eye phenotype in a *Drosophila* model of tauopathy by inducing autophagic pathways (Gentry et al., 2016). The evidence suggests that ROCK2 inhibition would have beneficial effects on both structural plasticities of neurons, through ROCK2’s prominent role in actin dynamics, and autophagic clearance of tau in AD (Figure 1B). Yet, critical questions remain regarding the precise molecular mechanisms involved and the putative interactions of ROCK2 signaling and mTOR pathways.

RNAi depletion of drug inhibition of ROCK2 reduces mTOR phosphorylation (Gentry et al., 2016); therefore, ROCK2 could influence autophagic pathways through mTORC1 and/or mTORC2. Whether ROCK2 signaling preferentially affects the mTORC1 or mTORC2 pathway remains to be determined. Moreover, mTORC2 can regulate autophagy through Akt which in turn mediates mTORC1 activity (Huang et al., 2015), thus ROCK2 could alter mTORC1 pathways through mTORC2. Finally, one of the most intriguing possibilities is that ROCK2 could regulate actin cytoskeleton dynamics by influencing mTORC2 activity (Figure 1C).

There is strong evidence in neurons that ROCK2 controls actin cytoskeleton rearrangement and dendritic structure through LIMK1 and cofilin (Rush et al., 2018; Henderson et al., 2019). However, could ROCK2 have similar effects on dendritic morphology by modulating mTORC2 activity? While mTORC2 does not seemingly affect organ size in skeletal muscle (Bentzinger et al., 2008), adipose tissue (Cybulski et al., 2009), or kidney (Gödel et al., 2011), brain architecture and neuron size are affected, suggesting a role for mTORC2 in CNS morphology (Angliker and Rüegg, 2013; Thomanetz et al., 2013). Conditional deletion of Rictor, a key component of mTORC2, in forebrain excitatory neurons of mice lead to decreased Ras-related C3 botulinum toxin substrate 1 (Rac1)-GTPase activity and reduced phosphorylation of downstream targets, including p21-activated kinase (PAK) and cofilin (Huang et al., 2013). This intersection of mTORC2 and Rho GTPase signaling could reflect a connection between ROCK2 and mTORC2 concerning actin re-organization in neurons, however additional experiments are warranted to fully elucidate the relationship. ROCK2’s emerging role as an autophagy regulatory protein, functioning through mTOR-related pathways, suggests exciting avenues of therapeutic potential for suppressing tau propagation in AD.

Work on the roles of ROCK1 and ROCK2 in non-neuronal cells in the brain has been more limited compared to neuron-centric studies. Not surprisingly, microarray and subsequent bioinformatics analyses of cultured primary murine astrocytes treated with the pan-ROCK inhibitor Fasudil demonstrated that inhibition of ROCKs altered transcription profiles associated with astrocytic morphology and motility, including genes involved in the actin cytoskeleton, extracellular matrix, and tight junctions. However, a portion of gene transcription alterations revealed that Fasudil induced pro-survival phenotypes in astrocytes that included excitatory amino acid transporter 2 and brain-derived neurotrophic factor as well as metabolic and anti-oxidative gene clusters (Lau et al., 2012). To this end, preclinical studies using Fasudil in rodent models of amyotrophic lateral sclerosis indicated that Fasudil treatment reduced infiltration of astrocytes but increased the presence of Iba1-positive microglial cells to sites of spinal cord degeneration. Parallel *in vitro* studies of microglial treated with lipopolysaccharides showed that Fasudil reduced secretion of proinflammatory cytokines and chemokines, including tumor necrosis factor-α, interleukin 6, and chemokine (C-C motif) ligands 2, 3 and 5 (Tonges et al., 2014). Additional work will be critical to better understand if and/or how ROCKs may affect autophagic pathways in non-neuronal cells in the brain.

## Author Contributions

AJW and JHH wrote the article. All authors contributed to the article and approved the submitted version.

## Conflict of Interest

The authors declare that the research was conducted in the absence of any commercial or financial relationships that could be construed as a potential conflict of interest.
